# Stereotypic behaviour predicts reproductive performance and litter sex ratio in giant pandas

**DOI:** 10.1038/s41598-020-63763-5

**Published:** 2020-04-29

**Authors:** Meghan S. Martin, Megan Owen, Nathan J. P. Wintle, Guiquan Zhang, Hemin Zhang, Ronald R. Swaisgood

**Affiliations:** 1 Institute for Conservation Research, San Diego Zoo Global, 15600 San Pasqual Valley Rd., Escondido, California 92027 USA; 2 PDXWildlife, 9233 SW Brier Pl., Portland, OR 97219 USA; 3 H-Corp Construction, 1626 SE Linn St., Portland, OR 97202 USA; 4 China Conservation and Research Center for the Giant Panda, Wolong, Sichuan 623006 P.R. China

**Keywords:** Behavioural ecology, Conservation biology

## Abstract

Breeding and welfare problems confront many conservation breeding programs. Stereotypies—repetitive, unvarying, functionless behaviours —are common abnormal behaviours that often arise in suboptimal conditions. While the role of stereotypies in welfare assessment is well studied, few investigations address the relationship between stereotypic behaviour and reproduction. We examined the correlation between stereotypic behaviour and reproductive performance in 101 giant pandas (*Ailuropoda melanoleuca*). High stereotyping males copulated more and produced more cubs, suggesting that highly sexually motivated males were prone to stereotypy but also had high reproductive competence. Female stereotypies were negatively associated with all reproductive measures closely tied to behavioural competence: high stereotyping females were less likely to copulate, less likely to mother-rear cubs, and—probably a result of poor maternal care—had lower cub survival. However, females that exhibited stereotypies were more likely to produce a cub, suggesting stereotypies are tied to behavioural but not physiological competence. High stereotyping female pandas also displayed strong and consistent bias toward production of female offspring while paternal relationship to sex allocation was the reverse. These results are consistent with stress-mediated sex allocation theory. Our findings raise concern about differential reproductive success among high and low stereotyping pandas, and possible genetic adaptation to captivity.

## Introduction

Animals in captivity often thrive and many species are known to survive and reproduce at rates exceeding their wild counterparts; on the other hand, many species fail to adapt to captivity and exhibit behavioural problems, reduced welfare, and poor reproduction and survival^1,2^. It is perhaps unsurprising, then, that breeding problems are common in many captive species in zoo and conservation breeding centres: reproductive behaviour and physiology may be compromised and/or infant mortality is high^3–7^. Indeed, the majority of managed breeding programs are not producing animals to replacement (i.e. where recruitment equals or exceeds mortality) and as few as 20% of recommended pairs successfully produce young before their next breeding and transfer plan^8,9^. Suboptimal housing and husbandry are often implicated as contributing factors to compromised reproductive performance^1,4,10–16^. Captive environments can also lead to domestication processes^17–19^ and abnormal behaviours^2,20,21^, which may contribute to reproductive failure.

Stereotypies—repetitive behaviours, unvarying in form, with no apparent goal or function—are common abnormal behaviours exhibited in captive-living animals^21,22^. While the performance of stereotypies often arises in suboptimal conditions associated with poor welfare, individuals that perform more stereotypies can demonstrate better welfare than less stereotypic individuals^20^. In zoo environments, where breeding uncommon species is a critical function, many forms of environmental enrichment have been developed to address welfare and reduce stereotypies^23,24^, with the understanding that enrichment may also enhance reproduction^11,25^.

The relationship between stereotypies and reproduction remains unclear and, given the large numbers of captive animals performing them (85 million domestic animals^20^, 10,000 zoo animals^4^) it is surprising how few studies address this question. Some studies demonstrate a negative relationship between stereotypies and reproduction^1,4,26,27^, whereas others have found that high stereotyping individuals show improved mating and reproductive success^28–30^. These contradictory results may stem from the complex relationship between stereotypies, stress, welfare, and reproduction^20,22^. Stereotypies may be positively associated with reproductive performance because (highly) stereotypic animals: 1) are more active, resulting in better physical fitness associated with higher fertility and/or fewer birthing problems^28^; (2) have found a coping response that reduces stress, boosting hypothalamic–pituitary–gonadal axis (HPG axis) function, and hence reproductive output^31^; or (3) are less prone to respond to the captive environment with a depression-like state^32,33^.The latter hypothesis is supported by evidence from several species indicating that individuals can respond to the environmentally induced stress either by performing frequent stereotypies or by becoming more inactive and unresponsive^31–33^. By contrast stereotypic animals may have less successful reproduction because (1) highly stereotypic animals have higher stress levels which are associated with suppressed HPG axis function^34^; or (2) highly stereotypic animals are more perseverative/behaviourally disinhibited, which compromises courtship and/or maternal care^35^. It is unclear which of these hypotheses may best explain the relationship between stereotypies and reproductive output, or even if they are mutually exclusive. Regardless, more empirical work is required to disentangle these complicated relationships between stereotypy and reproduction.

Further, an additional challenge facing conservation breeding programs is biased offspring sex ratio^36^. Sex allocation theory predicts that females in good condition should bias offspring production toward the sex with higher reproductive variance if greater investment leads to the development of higher quality offspring^37^. In polygynous systems, males have greater variation in reproductive success and maternal investment can produce more competitive males that leave more descendants. Evidence is accumulating in humans and animals indicating that females living in stressful, suboptimal conditions show bias toward production of females and this effect is often mediated by glucocorticoids^38,39^. Thus, where there is inter-individual variation in stress levels in captive environments—whether driven by external differences in living conditions or internal (e.g., personality) differences in response to stressors—we expect high stress levels to be associated with female-biased offspring production. Stereotypic behaviour performance may also be associated with birth sex ratios in two distinct ways. If high stereotyping individuals experience higher stress/glucocorticoids (more reactive to suboptimal environment), then high stereotypy performance should be associated with female-biased offspring production. If high stereotyping individuals experience lower stress/ glucocorticoid (better coping mechanisms in captive environment), then they should have male-biased offspring production. However, in a zoo conservation breeding setting, we are not aware of any test of this hypothesis even though the potential impact on the demographics and sustainability of a given population is large.

In addition, it is possible that stereotypic behaviour is related to paternal influences on offspring sex ratio. Growing evidence indicates that males can influence the sex of offspring, and that this influence is mediated by glucocorticoids through alterations of sperm quantity or quality^40–43^ and additional mechanisms^38^. Sperm quality is known to bias production of Y-chromosome-bearing sperm and bias sex ratios, with high glucocorticoids associated with female offspring production^44^. Thus, to the extent that stereotypy is associated with elevated glucocorticoids, stereotypy performance may also be associated with biased offspring sex ratios in males, yet this possible relationship appears to remain completely unexplored.

Despite the potential grave importance for conservation breeding programs, few studies have addressed the role of stereotypies in reproductive performance of any zoo-held or conservation-dependent species^27,45,46^. Here, we evaluate the relationship between stereotypy performance and reproduction in the giant panda, *Ailuropoda melanoleuca*. Giant pandas, once Endangered but now Vulnerable^47^, are held in a network of zoos and breeding centres as insurance populations and as a source for conservation translocations to re-establish wild populations. Stereotypic behaviours are relatively common in some captive giant panda populations but their occurrence can be mitigated with various forms of environmental enrichment^48–50^. Giant pandas have also experienced low reproductive rates in captivity^6,50,51^, although progressive husbandry management (e.g., twin swapping, milk collection^52^) and the application to breeding management of findings from behavioural research, have addressed many breeding problems and are associated with improved reproduction^50,53–55^. As a polygynous species, the panda is subject to predictions emanating from offspring sex allocation theory.

In this correlative study, we examined the relationships between performance of stereotypic behaviour and several measures of reproductive performance. Specifically, we examined whether the performance of stereotypies predicted intromission success, cub production, offspring sex ratio, maternal care, and cub survival.

Because we were unable to measure many of the factors potentially influencing stereroytpy performance, we cannot make predictions based on stress, physical fitness, emotional state, or behavioural perseverance. Instead, our results can be consistent or inconsistent with several of the hypotheses for the relationships between stereotypy and reproduction. If, for example, highly stereotypic individuals have better reproductive performance, we may conclude that these individuals have better physical fitness, that they experience less stress/have lower glucocorticoids, or respond to a stressful environment with stereotypy rather than a depression-like state. By contrast, if less stereotypic individuals have better reproductive performance, then we can conclude that they experience less stress/have lower glucocorticoids or they are less perseverative. Interpretation of these patterns may be enhanced by outcomes for offspring sex ratio. If offspring production is sex-biased, the only known causal link relating to the above hypotheses is stress. If highly stereotypic individuals produce female-biased litters, this suggests that they also have higher glucocorticoids, confirming previous studies showing a correlation between glucocorticoids and stereotypy performance in female pandas^56^. If highly stereotypic individuals produce male-biased litters, this suggests they have lower glucocorticoids and experience less stress.

Yet another way we attempt to gain insight into the possible causality in this correlational study was to examine how sex and season influenced stereotypic performance. We reasoned that different stereotypy patterns among males and females across breeding and non-breeding seasons may help reveal underlying motivation in a reproductive context. If reproductive motivation underlies performance of (some) stereotypies, we expect stereotypy performance to change during the breeding season for males, females or both.

## Results

### Seasonal influences on stereotypic behaviours

To provide sex-specific insights into the motivational basis for stereotypy performance in pandas, we first examined how stereotypies changed across the breeding and non-breeding seasons using GLMMs.

**Figure 1:**
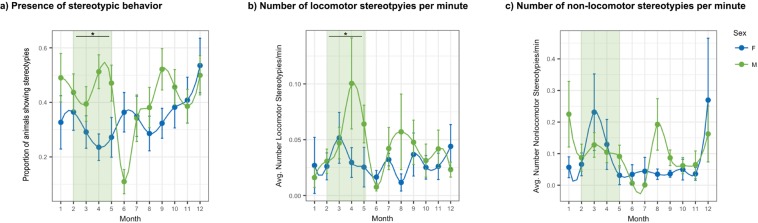
Relationship between sex and season and stereotypic performance. The a) presence of stereotypic behaviour, b) number of locomotor stereotypies, and c) number of non-locomotor stereotypies for male (green) and female (blue) giant pandas across the year. Breeding season typically lasts from February to May. Asterisks indicate significant differences at p < 0.05 for GLMM interaction term of season*sex, adjusted for inflated Type I errors using the Benjamini-Hochberg procedure. Error bars depict standard error.

The presence of stereotypic behaviour varied across season (Fig. 1a; GLMM; β = 0.27, *Wald χ*^2^ = 2.27, *p* = 0.02) and between sexes (Fig. 1a; GLMM; β = 0.52, *Wald χ*^2^ = 2.02, *p* = 0.04). The interaction term between sex and season was also significant, with male giant pandas showing a higher occurrence of stereotypic behaviour than female giant pandas during the breeding season (Fig. 1a; GLMM; β = −0.23, *Wald χ*^2^ = −1.44, *p* = 0.05). Males also displayed more locomotor stereotypies than did females (Fig. 1b; GLMM; β = 0.02, *Wald χ*^2^ = 1.51, *p* = 0.01) and displayed significantly more locomotor stereotypies within the breeding season (Fig. 1b; GLMM; β = −0.18, *Wald χ*^2^ = −2.55, *p* = 0.01). There was no difference in non-locomotor stereotypic behaviour across seasons, between sexes, or with the interaction term of season*sex (Fig. 1c).

### Female reproductive performance

**Figure 2:**
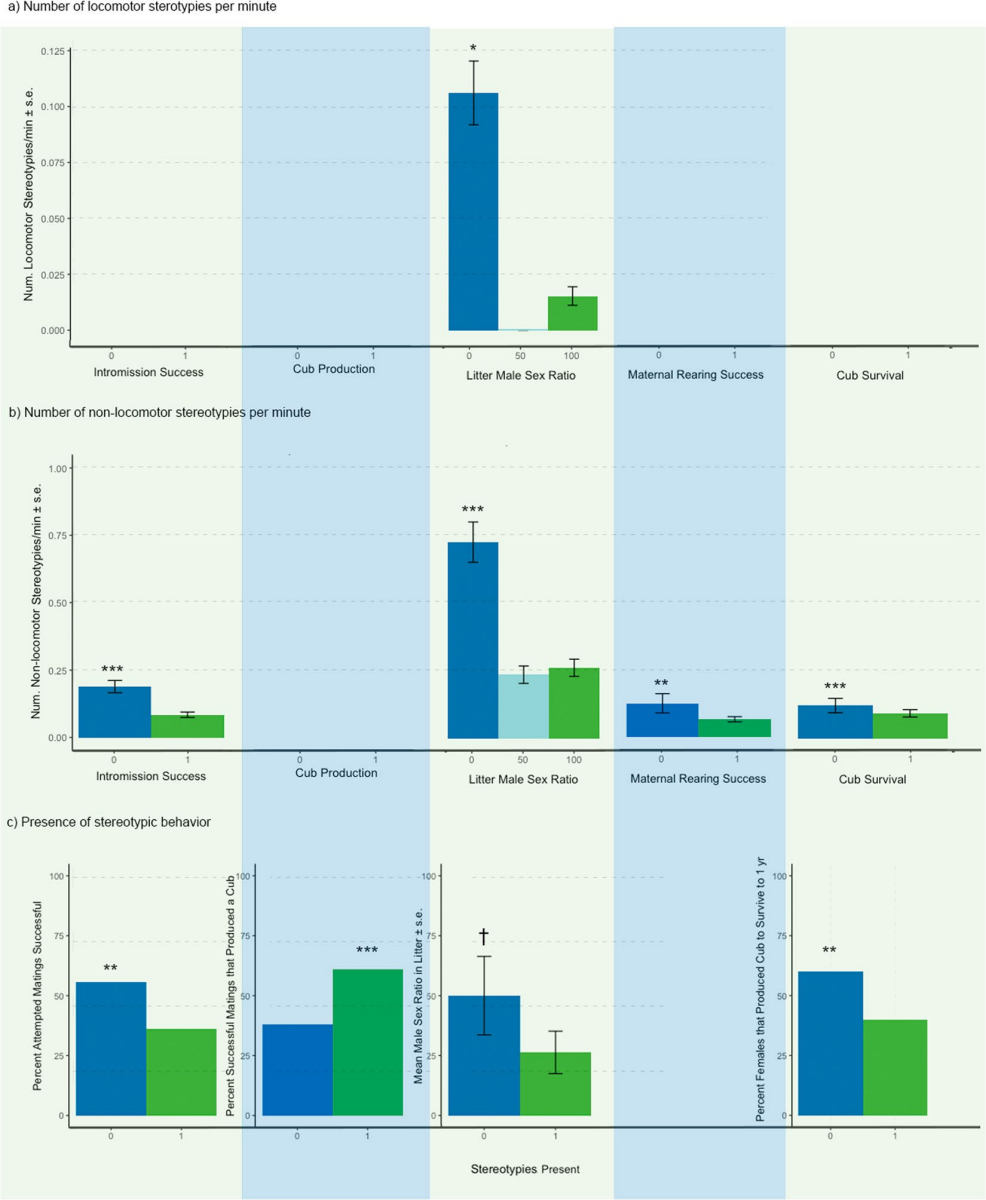
Significant relationships between stereotypies (mean + SE) and reproductive performance in female giant pandas. Asterisks indicate significant differences (*p < 0.05; **p < 0.01; ***p < 0.001), adjusted for inflated Type I errors using the Benjamini-Hochberg procedure.

Several measures of reproductive performance were associated with stereotypic behaviour in female giant pandas. We present all statistical results in Table 1 and present significant findings in Fig. 2 and in the text below.

**Table 1:** GLMM analyses evaluating relationship between stereotypies and measures of reproductive performance in female giant pandas.

	Frequency of locomotor stereotypies (β, *Wald χ*^2^, *p*)			Frequency of non-locomotor stereotypies (β, *Wald χ*^2^, *p*)			Stereotypy Present 1,0 (β, *Wald χ*^2^, *p*)		
Successful intromissions (1/0; N = 118)	−2.11,	−1.23,	0.22	**−8.30**,	**−25.92**,	**<0.001**	**−0.02**,	**−22.88**,	**0.004**
Cubs produced (1/0; N = 118)	18.42	1.53,	0.13	4.18,	0.90,	0.37	**0.48**,	**3.24**,	**0.001**
Number of cubs (N = 57)	−0.79,	−0.53,	0.59	−0.21,	−0.24,	0.81	−0.04,	−0.12,	0.91
Male sex ratio (N = 57)	**−9.83**,	**−1.28**,	**0.03**	**−1.22**,	**−3.66**,	**<0.001**	*−0.71*,	*−1.81*,	*0.07*
Cubs mother-reared (1/0; N = 57)	10.74,	2.71,	0.57	**−38.99**,	**−2.52**,	**0.01**	−0.01,	−0.18,	0.85
Cubs survived to one year of age (1/0; N = 57)	−44.66,	−1.10	0.27	**−27.97**,	**−4.11**,	**<0.001**	**−28.28**,	**−3.02**,	**0.002**

Significant relationships *p* < 0.05 (adjusted for inflated Type I errors using the Benjamini-Hochberg procedure) are indicated in bold type while trends *p* < 0.08 are indicated in italics.

To evaluate the relationship between stereotypies and breeding behaviour, we examined intromission success. Females that showed low levels of non-locomotor stereotypies (Fig. 2a) and that never displayed stereotypic behaviours during our observations (Fig. 2c) were significantly more likely to achieve intromission than females that did display stereotypies.

To examine the relationship between stereotypies and reproductive output, we evaluated cub production, number of cubs in a litter, maternal rearing, and cub survival. Females that displayed stereotypies (presence/absence) were more likely to produce a cub and have the cub survive to one year of age than females with no stereotypies (Fig. 2c). However, among stereotyping females, those that successfully reared at least one cub displayed fewer non-locomotor stereotypic behaviours than those that did not successfully rear a cub, and females that successfully had at least one cub survive to one year of age also displayed fewer non-locomotor stereotypic behaviours (Fig. 2b). Litter size was not affected by the frequency or presence of stereotypies exhibited by female giant pandas.

Finally, we evaluated whether stereotypy performance was related to sex ratios of offspring produced. Females that produced female biased offspring sex-ratios displayed more frequent locomotor (Fig. 2a) and non-locomotor stereotypies (Fig. 2b). Post hoc comparisons using the Tukey HSD test indicated that litters containing 0% males had mothers that performed both locomotor and non-locomotor stereotypies at a significantly higher rate than litters containing 50% males and 100% males (p < 0.05; Fig. 2a,b). There was no difference in stereotypy performance between 50% and 100% (p > 0.05). Females that performed stereotypies showed a trend toward producing female-skewed litters compared to females that were not observed performing stereotypies (Fig. 2c).

### Male reproductive performance

**Figure 3:**
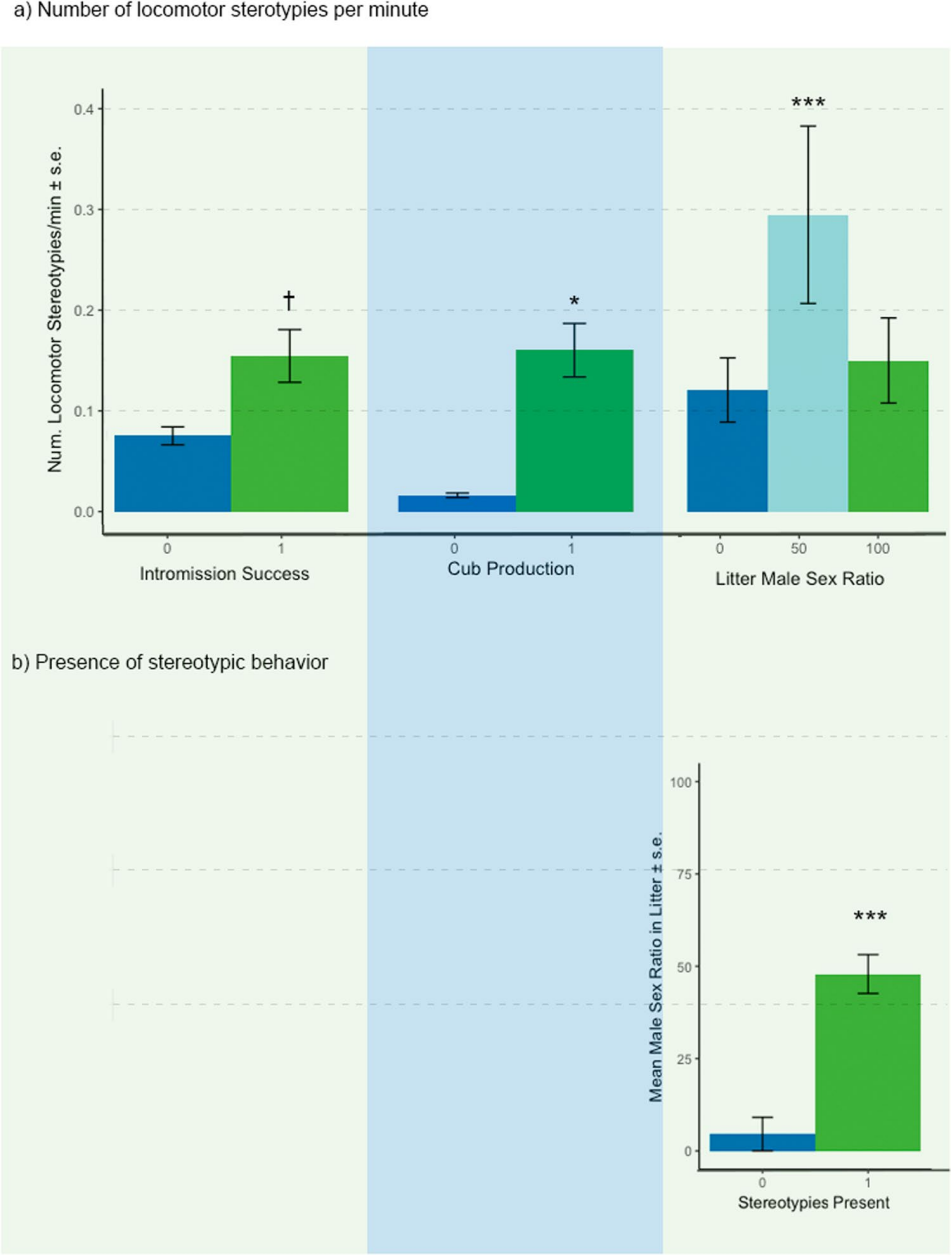
Significant relationships between stereotypy measures (mean + SE) and reproductive performance in male giant pandas. Asterisks indicate significant differences from GLMMs (*p < 0.05; **p < 0.001; ***p < 0.001), adjusted for inflated Type I errors using the Benjamini-Hochberg procedure.

Male stereotypy performance was associated with several measures of reproductive performance (Table 2, Fig. 3). Only one stereotypic variable was associated with intromission and cub production in males: males displaying more frequent locomotor stereotypies were more likely to achieve successful intromissions (although this was a non-significant trend) and significantly more likely to produce cubs (Fig. 3a).

**Table 2:** GLMM analyses evaluating relationship between stereotypies and measures of reproductive performance in male giant pandas.

	# Locomotor stereotypies/min (β, *Wald χ*^2^, *p*)			# Non-locomotor stereotypies/min (β, *Wald χ*^2^, *p*)			Stereotypy Present 1,0 (β, *Wald χ*^2^, *p*)		
Successful intromissions (1/0; N = 213)	*1.11*,	*1.42*,	*0.06*	−0.63,	−0.50,	0.62	−0.01,	−1.64,	0.10
Cubs produced (1/0; N = 92)	**9.88**,	**2.23**,	**0.03**	−0.44,	−1.11,	0.27	0.01,	1.30,	0.19
Number of cubs (N = 71)	0.10,	0.48,	0.63	0.14,	0.87,	0.38	0.19,	0.70,	0.48
Male sex ratio (N = 71)	**0.90**,	**5.82**,	**<0.001**	0.08,	0.32,	0.74	**1.43**,	**4.15**,	**<0.001**

Significant relationships *p* < 0.05 (adjusted for inflated Type I errors using the Benjamini-Hochberg procedure) are indicated in bold type while trends *p* < 0.08 are indicated in italics.

Males exhibiting locomotor stereotypies were less likely to produce two-cub litters (24.8% of produced litters were twins) than non-stereotyping males (51.1%) but if they produced twins, they were more likely to have mixed sex litters (60% of twin litters vs. 34.8% respectively; Fig. 3a). Post hoc comparisons using the Tukey HSD test indicated that males with twin litters containing 50% males showed significantly more locomotor stereotypes than studs with litters containing 0% males (p = 0.03; Fig. 3a) but there were no significant differences in stereotyping behavior between 50% and 100% or 100% and 0% male sex ratio litters (p > 0.05). Male giant pandas that performed stereotypies had significantly more male-skewed litters compared to males that were not observed performing stereotypies (Fig. 3b).

### Discussion

Our findings provide the first empirical evidence that stereotypy performance is related to reproduction in a conservation breeding program. The substantially different patterns of stereotypy performance in male and female pandas are intriguing. Both sex and breeding season were influential predictors of some measures of stereotypy performance, suggesting that the underlying motivation of some stereotypies may be driven or exacerbated by sexual motivation. Interestingly, female locomotor stereotypies decreased during the breeding season and male locomotor stereotypies increased. This observation is consistent with the substantial increase in male mate searching behaviour seen in the mating season in the wild contrasting with the female’s more sedentary mating tactics^57,58^. Although there is a relatively brief increase in locomotor behaviour in female pandas just before peak oestrus^48^, male pandas dramatically increase their ranging behaviour in search of females over a sustained period. Males also congregate around females and compete with other males for reproductive access to the female^57,58^ while females typically position themselves in a tree to await the outcome of male-male competition. Females also have a short breeding window (typically fertile for 2–3 days), while males remain sexually active for several months, which are characterized by increased ranging behaviour in search of females. Although female choice is also important^54,59,60^, male reproductive behaviour appears highly motivated and involves more physical activity and heightened testosterone^57^.

Many stereotypies appear to arise from frustrated motivation to perform certain behaviours seen in nature—such as foraging, ranging or mate searching—that cannot be fully expressed in the confines of the captive environment^22,61–63^. We can thus plausibly conclude that the pattern of increasing stereotypies during the mating season in males is largely the result of frustrated motivation by males to access neighbouring females. Indeed, this is consistent with our own observations of male pacing and head tossing occurring primarily along the enclosure wall adjoining females, particularly if they are in oestrus. If so, our findings suggest that the relatively small enclosures in which these pandas were housed meet more of the males’ behavioural needs outside of the mating season, when males do not have the additional motivation to search for mates. Outside the mating season, the level of stereotypic behaviour is similar between males and females, suggesting that other, non-reproductive, behavioural needs may motivate these stereotypies. These may include feeding and foraging behaviour, as suggested by observations that anticipation of delivery of provisioned food is associated with stereotypy performance in pandas^49^ and other species^19^.

Our analyses of the relationships between stereotypies and reproductive performance reveal several important insights. While locomotor stereotypies were poor predictors of most reproductive outcomes in female giant pandas, there were a number of differences between females that stereotyped and those that did not, and as a function of frequency of non-locomotor stereotypy performance. Females that were high stereotyping were less likely to copulate, but females that exhibited stereotypies were more likely to produce a cub, so results are mixed for female reproductive success. Stereotypy performance was also negatively associated with maternal rearing and cub survival, suggesting that high stereotyping females provided less competent maternal care. Perhaps stereotypy performance can interfere directly with female reproductive behaviour—as seen in other species^61^—or female stereotypies may be associated with behavioural dispositions that inhibit mating (e.g., timidity, behavioural inflexibility^2^, and/or inability to cope with change and challenge). Individual variation in the prevalence of stereotypies may reflect one of several potential non-mutually exclusive realities in captive environments^22^: (1) they may develop in individuals that perceive the environment as more aversive and therefore experience more stress; (2) they may be a sign that the individual is coping better with the captive environment, as stereotypy performance can reduce indices of stress; (3) they may be a ‘scar’ from earlier development having lasting effects on central nervous system organization resulting in perseverance of stereotypy performance in other, more suitable environments; or (4) they may reflect individual variation in the propensity to respond to an aversive environment with depression-like responses vs. stereotypy performance. This uncertainty or multi-modal response to suboptimal environments renders difficult interpretation of our stereotypy and reproduction findings. Future research should address this with an *a priori* experimental design to manipulate a single variable (e.g., situations associated with high and low stress levels, with independent measures of stress) to elucidate causal mechanisms behind our correlations.

The pattern of positive and negative association of stereotypies with reproductive performance in female pandas is interesting to contemplate. We found a negative correlation between stereotypic behaviour and reproductive measures more closely tied to behavioural competence: high stereotyping females were less likely to copulate, were less likely to mother-rear their cub, and—most likely as a result of poor maternal care—had lower cub survival. Indeed, pacing behaviour has been observed in conjunction with maternal rejection of offspring in giant pandas^64^. These results suggest that stereotypy performance in female giant pandas is strongly tied to behavioural competence and that individuals performing more stereotypies are less able to perform important behaviours that are part of their natural behavioural repertoire associated with mating and maternal care. That is, these high stereotyping females are poorly adapted to captivity, and the captive environment may be selecting against females that display high stereotypy levels. Alternatively, stereotypies may inadvertently signal to males a lack of sexual receptivity or may diminish attractiveness^1^. However, high stereotyping females produced more cubs, indicating that they have higher fertility levels or are better able to sustain pregnancy than are low stereotyping females. This result suggests that low stereotyping females may be physiologically compromised. A plausible explanation is that high stereotyping females respond more negatively to the captive environment—due to underlying behavioural dispositions or personality types—but stereotypy performance reduces stress and its attendant consequences for reproduction^65^. Alternatively, if low-stereotyping females were less active and overweight, fertility may have been compromised^28^ or low-stereotyping females may be reflection of a depression-like state^33^ that also negatively influenced fertility.

Our findings for female panda reproduction and stereotypies show parallels and divergences from studies with other species. Several studies have also demonstrated a positive effect of stereotypy on female offspring production^18,22,28–30^, but at least one study has demonstrated a negative effect of stereotypy performance on conception rates^26^. Evidence for compromised maternal care among high stereotyping individuals is also mixed, with some studies demonstrating faster growing and/or better surviving offspring for high stereotyping females^18,28,29^ and other studies showing negative effects of stereotypy on maternal care or offspring survival^11,66,67^. Thus, similar to our results, there appears to be more support across species for positive effects of stereotypies on offspring production by females, but negative effects on more directly behaviourally mediated aspects of reproduction, such as maternal care of offspring. Variation and inconsistencies in results are no doubt the result of many complexities, including the timing of stressors and stereotypies with regard to the reproductive cycle and the potential increase in physical fitness of stereotyping females which may support cub production.

Male pandas, unlike females, appear to be motivated to perform stereotypies in part due to frustrated reproductive motivation. Unable to exercise their natural behaviour of searching and courting females, they develop stereotypies—largely pacing at the edge of their enclosures adjacent to females—because their appetitive behaviour for courtship and mating cannot reach the consummatory phase. Thus, it is plausible that high stereotyping males have the highest sexual motivation, explaining why males that engage in more locomotor stereotypy—which peaks during the mating season—copulate more and produce more cubs. Most parsimoniously, high reproductive performance and high rates of stereotypies in male pandas may both be the product of high levels of sexual motivation, and there may be no direct causal relationship between stereotypies and reproduction. Alternatively, if stereotypy performance reduces stress, it may be causally related to reproduction. Stress is known to negatively affect vertebrate male reproduction through reduced androgen levels, testis size, sperm production and spermatogenesis^65,68–70^. While males do perform non-locomotor stereotypies and engage in stereotypic behaviour outside of the mating season, we found no evidence for a relationship between non-locomotor stereotypy and reproductive performance.

Our most robust findings indicate that stereotypies are closely tied to maternal birth sex ratio. As outlined in the introduction, as a polygynous species, sex allocation theory^37^ predicts that females in good condition (normal body weight with some fat stores) should produce male-biased litters because investing in male offspring when resources are plentiful increases reproductive fitness when male offspring sire more offspring than female offspring. Environmental stressors—such as limited resources, social competition and crowding—may influence sex ratio adjustment through activation of the hypothalamic–pituitary–adrenal axis and glucocorticoid production^38,71^. Although results are somewhat mixed, most studies indicate that stressors increase female-biased offspring skew^38,71^, and controlled experimental manipulation increasing glucocorticoids (stress hormones) also have been shown to increase female sex ratio in mammals^72^ and birds^73^, although results for some species link stressors to male-biased sex ratios^74,75^.

These findings suggest that stressors in captivity may influence birth sex ratios. Although captive females held in zoos presumably have ample nutritional resources, and therefore are predicted to have male-biased offspring production, cross-species analysis indicates that birth sex ratios on the whole are near parity^36^. It is possible that stressors in captivity offset any nutritional advantages over wild counterparts and play a role in maintaining balanced sex ratio. Our findings indicate that high-stereotyping female pandas had strong and consistent bias toward production of female offspring across all stereotypy measures we analysed. To the extent that stereotypy performance is a reliable indicator of stress (see above), our findings are consistent with the hypothesis that stress favours female-biased sex ratios. This interpretation requires that corticoids be higher in stereotyping pandas, which has been demonstrated previously^56^. An alternative explanation is that low-stereotyping females were less active and therefore had more body fat, which is associated with the male-biased offspring production.

We also found strong evidence for a relationship between paternal stereotypies and offspring sex ratios. Although sex allocation theory has been applied almost exclusively to mothers, fathers stand to benefit from adaptive sex allocation as much as mothers^76^. Erroneous assumptions that male X/Y chromosome-bearing sperm cells were genetically controlled during meiosis have been re-evaluated in light of growing evidence that male mammals in particular have the ability to adjust sex ratio in sperm adaptively^76^. In contrast to female pandas, stereotypic males produced litters biased toward males. This is consistent with our interpretation that much of male stereotypic behaviour is motivated by thwarted reproductive behaviour. Highly sexually motivated (and stereotyping) males may, under typical natural conditions, have better body condition, win intra-male competition, and have higher testosterone levels. It is also possible that female giant pandas are altering sex ratio through post-fertilization mechanisms related to perception of male quality^77^ (also for review see Navara Chapter 4^78^). Although the hormonal regulation of paternal sex allocation is not yet known, elevated testosterone is a plausible candidate^76^. Our findings thus indirectly support the growing body of work supporting adaptive paternal sex allocation.

These findings may have considerable implications for the management of captive breeding programs. If sex ratios of captive breeding populations are skewed due to stereotypic behaviour (mediated by stress hormones), there may be consequential imbalance favouring one sex over the other. Although overproduction of females is not necessarily detrimental for population growth in polygynous species, we need to understand what factors cause sex ratio adjustment. For example, if stress does lead to the overproduction of females, husbandry practices that mitigate stress may inadvertently lead to male-biased offspring production. Further research is now required to disentangle the complex relationships between stereotypies, stress, environmental factors, husbandry, reproduction, and sex ratio allocation to guide management decisions.

In addition to these immediate concerns related to reproductive output, our findings raise the possibility of inter-generational adaptation to captivity, or domestication. We documented differential reproduction on the basis of stereotypy performance in pandas, which may indicate selection for phenotypes related to stereotypy. Previous findings that pandas mate assortatively based on personality traits that include stereotypy^60^ indicate that mate preferences may exacerbate the process of stereotypy-related domestication. Recent evidence for rodent species has shown that stereotypy performance can be favoured in captivity, with markedly higher reproductive output^29^ and directional evolutionary increase in stereotypy performance in just ten generations^18^. In addition to artificial selection for (or against) stereotypies, genetically correlated morphological and behavioural traits have the potential to be irrevocably altered as the result of adaptation to the captive environment and relaxation of traits important for survival in the wild. While in some cases this evolutionary change may improve adaptation to captivity, they can have considerable negative consequences for conservation breeding and translocation programs, where preservation of wild genotypes is an important goal^19^. To avoid this outcome, we recommend managing the breeding and maternal care environments to better support animals that are reproducing poorly in captivity, ensuring a diversity of behaviours are expressed and maintained in the population.

## Methods

### Ethics

All applicable international, national, and/or institutional guidelines for the care and use of animals were followed. Animal care and use guidelines of the American Society of Mammologists (Institutional Animal Care and Use Committee 1998; San Diego Zoo Assurance #: 15-003 and #17-003) were followed by all facility operators. The procedures used in the research did not affect the housing, diet or management of the animals and comply with the law of the People’s Republic of China.

### Study species and husbandry

We studied giant pandas at the Wolong and Bifengxia breeding facilities of the Chinese Conservation and Research Center for the Giant Panda in Sichuan Province, China. We collected data during the breeding season (February–May) and non-breeding season (June–January) of 1997–2002 at Wolong and 2012–2016 at Bifengxia.

During the breeding season at Bifengxia (2012–2016), giant pandas were housed individually in concrete walled, open-air enclosures (8 m x 25 m) that contained an indoor enclosure area (3 m x 8 m; for detailed housing information see Martin-Wintle *et al*.^54^). Pandas had visual, auditory and olfactory access to one or two neighbouring pandas through four interaction windows on each side of the enclosure. At Wolong giant pandas were housed in similar, but smaller (10 m x 10 m), enclosures with three interaction windows on each side of the enclosure, and access to an inner bedroom (3 m x 5 m; for detailed housing information see Swaisgood, Lindburg & Zhou, 1999^79^). During the non-breeding season, giant pandas were either housed in the enclosures described above or larger (range = 93m^2^–10,000m^2^) more naturalistic enclosures. These enclosures had varying access to neighbouring pandas but typically had more limited visual, olfactory and acoustic access to neighbours. All enclosures contained various forms of environmental enrichment (e.g. climbing platforms, water features, trees, etc.) and giant pandas were exposed to natural light conditions. It is important to note that current housing conditions do not adequately reflect housing histories for individuals, and some subjects will have experienced smaller, more impoverished enclosures and varying husbandry practices for a portion of their development. Unfortunately, these and other differences in developmental histories are not sufficiently known for inclusion in analysis. Giant pandas were fed a diet of local bamboo supplemented with bread, high-fibre biscuits, carrots, and apples.

### Procedure

During the years of 1997–2002 and 2012–2016 we observed the behaviour of 101 adult giant pandas (29♂, 72♀; 6 years or older). We conducted focal, all-occurrence sampling year-round during 90- minute sessions approximately four times per month. Observations were conducted in a balanced fashion across morning (07:00–11:30) and afternoon (13:30–16:00) sessions. Animal husbandry activities sometimes interrupted observations, and resulting sessions ranged from 16–90 minutes in length (mean = 52.17 min ± 12.6 SD) and we obtained 2–45 sessions (mean = 9.7 ± 3.9) per individual. Stereotypy criteria required the rigid performance of the same behaviour form three or more times in a row and/or was embedded in a routine in which it is repeated at least three times (Supplementary Table S1). To obtain behavioural frequencies, behavioural bouts were separated by >5 seconds performing a different behaviour. All observers were tested for interobserver reliability with an experienced observer to 85% or higher agreement reported by the ICC2 using the *ICC* function in the *psych* library in R. To accommodate for the varying duration of observational periods, behavioural observations for each animal were standardized to behaviours per minute for frequency data (events). We controlled for overall activity by dividing all behaviours by the total minutes giant pandas were active (i.e. not sleeping or resting).

To determine the seasonal difference between sexes in stereotypic behaviour, we averaged each individual’s stereotypies within each month across years. On average there were 44 unique individuals to compare in each month. To compare stereotypic behaviour within the year to reproductive success in that same year, and because we found seasonal differences in stereotypic behaviour, behavioural observations were averaged per individual across the nonbreeding season (June-January) within each year and used to classify individuals as stereotypic or non-stereotypic. We chose the non-breeding season because this time period represented the longer of the two seasons and additionally, it captures a long-term, chronic picture of stereotypical behaviour. To compare the frequency of locomotor and non-locomotor stereotypic behaviour within the year to reproductive success in that same year, behavioural observations were averaged per individual across the entire year. To reduce the number of stereotypic variables for analysis, we summed all locomotor and non-locomotor variables into two discrete variables (Table 3). We made this distinction because they differ categorically in form and because we hypothesized they may be motivationally distinct (cf. Polanco *et al*.^80^). Locomotion is the main behaviour animals use to avoid aversive stimuli or gain access to resources, thus pacing stereotypies may provide a window into motivation to leave the enclosure. The origin and motivational basis for non-locomotor stereotypies seem less clear, although some may be associated with feeding motivation (e.g., weave and sway often occurred at locations where caretakers deliver food and tongue-flicking often occurred for long periods following eating). Thus, we categorized these behaviours simply as non-locomotor because they do not involve locomotion and it is difficult to reliably categorize them further based on possible motivational basis.

**Table 3:** Giant panda stereotypic behavioural indices.

Behaviour	Definition
Frequency of locomotor stereotypy behaviours (behaviours/minute)	Sum of frequencies of pacing and quasipacing stereotypies within an observation session divided by the total minutes the panda was active during the observation period.
Frequency of non-locomotor stereotypy behaviours (behaviours/minute)	Sum of frequencies of pirouette, head toss, self-bite, somersault, weave, sway, tongue flick, sit up, paw suck, cage climb, regurgitation of food, rolling, licking food, paw tap, and scratching divided by the total minutes giant pandas were active during the observation period.
Presence of stereotypic behaviour (1/0)	The presence of at least one bout of stereotypic behaviour was observed across all observations. For analysis of seasonal relationship with stereotypy, the unit of analysis was presence-absence within an observation session. For analysis as a predictor variable on reproductive outcomes, the unit of analysis was the presence/absence of stereotypy observed in the individual (i.e., stereotyping and non-stereotyping individuals).

See Supplementary Table S1 for definitions of each individual behaviour listed under “Definitions”.

During the breeding season (February-May) we paired giant pandas during a female’s oestrus following genetic recommendations based on mean kinship analysis to minimize breeding. Mating introductions were conducted by breeding managers who evaluated sexual and aggressive motivation to determine when to pair and separate animals (details in Swaisgood *et al*.^81^ and Martin-Wintle *et al*.^54^). As a fail-safe, female giant pandas were often artificially inseminated following natural breeding. If paternity was in question, the CCRCGP established the paternal identity using DNA obtained from hair samples. DNA was amplified using the polymerase chain reaction to analyse microsatellite loci after the methods of Zhang *et al*.^82^. All cubs included in this study had confirmed paternity and only cubs conceived from the natural mating events were used in analyses.

Reproductive performance variables for each year for each subject included: intromission success (1/0), litter production (1/0), sex ratio of the litter, whether females maternally raised at least one cub (1/0), and whether at least one cub survived to one year of age (1/0). Maternal rearing of a cub indicated the likelihood of abandonment of the cubs early (usually within the first day to week) whereas cub survivorship was a reflection of overall quality of maternal care in the first six months as well as cub behavioural and immunocompetence from 6 months to one year of age upon weaning. Reproductive variables were subsets of the successful events from upstream reproductive variables (e.g., we analysed litter production only among individuals that had gained successful intromissions and cub survival analyses among females that maternally reared cubs). Stereotypic data for the focal animal was matched to the breeding data collected for that same year.

### Data analysis

We analysed 118 breeding attempts resulting in 57 litters for females, and 213 breeding attempts resulting in 71 litters for males. To evaluate the influence of sex and season on stereotypy performance, we averaged individuals within the breeding season (February – May) and non-breeding season (June – January) across years and used GLMMs with sex and season and their interaction as explanatory variables, individual ID as a random factor, and the stereotypy variables as response variables.

To evaluate the ability of stereotypies to predict reproductive performance, we used the *lme4* package in R. We ran GLMMs to examine the relationship between each of the stereotypy explanatory variables and the reproductive performance variables. We included provenance (wild / captive) and age (in years) as fixed factors in order to account for reproductive and stereotypic parameters which may change across an individual’s lifetime and/or with previous life experiences. We included individual ID and facility ID as random factors to account for variation in reproductive success across individuals and facilities. Logistic regression with a logit-link function was used for the binary reproductive performance variables intromission success, cub production, maternal rearing, and cub survivorship to one year. For litter sex ratio we used the above GLMM format with the natural log of the total litter size as an offset variable and number of male cubs in a litter as the response variable with Poisson distribution. We performed post-hoc tests to determine which sex ratio groups were significantly different using the *glht* function in the *multcomp* package.

The above analyses resulted in a large number of statistical tests for each research question, therefore we employed the Benjamini−Hochberg^83^ procedure using the *p.adjust* function with method set to “fdr” in the *stats* package in R to address family-wise error rates. All reported p-values reflect this adjustment and meet the criteria for significance. When examining all possible paired comparisons in our analysis of sex ratios, we used Tukey’s HSD to provide a conservative adjustment to p-values. All statistical significance tests done in the manuscript were two sided. All analyses were performed in R Studio (Version 1.0.143; R Studio Inc. 2009–2016; R Version 3.3.3).

## Supplementary information

Supplemental Table S1.

## Data Availability

The datasets generated during and/or analysed during the current study are available from the corresponding author on reasonable request.
