# Supplementary material for: Stereotypic behaviour predicts reproductive performance and litter sex ratio in giant pandas
Source: Sci Rep. 2020 Apr 29;10:7263. doi: 10.1038/s41598-020-63763-5 (PMC7190838; doi:10.1038/s41598-020-63763-5)
Supplement: Supplementary file 1 — Supplemental Table S1. [file 41598_2020_63763_MOESM1_ESM.docx]

Stereotypic behaviour predicts reproductive performance and litter sex ratio in giant pandas. Meghan S. Martin, Megan Owen, Nathan J.P. Wintle, Guiquan Zhang, Zhang Hemin, Ronald R. Swaisgood.

**Supplemental Table S1. Stereotypic behavior ethogram for the giant panda.** Stereotypy definition criteria require the rigid performance of the same behaviour form three or more times in a row and/or is embedded in a routine in which it is repeated at least three times. For example, pirouettes and head-tosses may occur repeatedly while locomoting, but separated by bouts of locomotion.

| **Behaviour** | **Definition** |
| --- | --- |
| *Locomotor stereotypies* | |

| Stereotypic  pacing | Back and forth, or perimeter travel in a repetitive, sustained, locomotor pattern. |
| --- | --- |
| Quasi-stereotypic  pacing | Same as stereotypic pacing above, except animal need not take the same path 3 or more times in a row. Any pacing in which a predictable pattern emerges. There may be variations in the routine or the animal may alternate between a limited number of travel paths. |
| *Non-locomotor stereotypies* | |
| Pirouette | Stands on hind legs and spins at least 90 degrees (hindquarters may not leave ground in some cases). |
| Head-toss | Animal abruptly lifts head upward and/or to the side in a swinging movement. |
| Self-biting | Animal bites itself repeatedly. |
| Stereotypic somersault | Full or truncated somersault. Full somersault involves placing head on ground and pushing hindquarters over the head in a complete revolution. Truncated somersault involves placing head to ground as if to do a somersault and touching shoulders to the ground but does not complete action. |
| Weave | Animal undulates the front portion of the body back and forth repetitively, somewhat reminiscent of a fish’s swimming movements. |
| Sway | Animal swings only the head back and forth but does not have undulating movements associated with weaving. |
| Tongue flick | Animal sticks tongue out repetitively in a licking movement; not associated with feeding or grooming the area around the mouth after feeding. |
| Sit-up | Animal sits down, lies on back (moving the head away from the toes) then sits upright (moving head toward the toes) repetitively. |
| Paw suck | Repetitive, sustained sucking of the toes/paw, not associated with grooming. |
| Cage climb | Animal stands bipedally and sways or makes climbing motions, as if attempting to escape. |
| Stereotypic regurgitation | Animal vomits and re-ingests/licks vomit repeatedly. |
| Stereotypic roll | Rolls from side to side on the ground repetitively. |
| Stereotypic licking of food item | Animal interrupts feeding bout with a long bout of licking of food item in which consumption of food does not occur. |
| Paw tap | Animal taps paw repeatedly while limbs are relaxed and animal is stationary. |
| Stereotypic scratching | Animal scratches itself repeatedly; form is rigid and scratch directed to same location on body (not grooming). |
